# Supplementary material for: Synaptotagmin 1 oligomerization via the juxtamembrane linker regulates spontaneous and evoked neurotransmitter release
Source: Proc Natl Acad Sci U S A. 2021 Nov 22;118(48):e2113859118. doi: 10.1073/pnas.2113859118 (PMC8694047; doi:10.1073/pnas.2113859118)
Supplement: Supplementary File [file pnas.2113859118.sapp.pdf]

PNAS
www.pnas.org

Supplementary Information for

Synaptotagmin 1 oligomerization via the juxtamembrane linker regulates spontaneous and evoked neurotransmitter release.

Kevin C. Courtney, Jason D. Vevea, Yueqi Li, Zhenyong Wu, Zhao Zhang and Edwin R. Chapman

Edwin R. Chapman
Email: chapman@wisc.edu

This PDF file includes:

- Figures S1 to S14
- Tables S1 to S2
- Supporting Materials and Methods

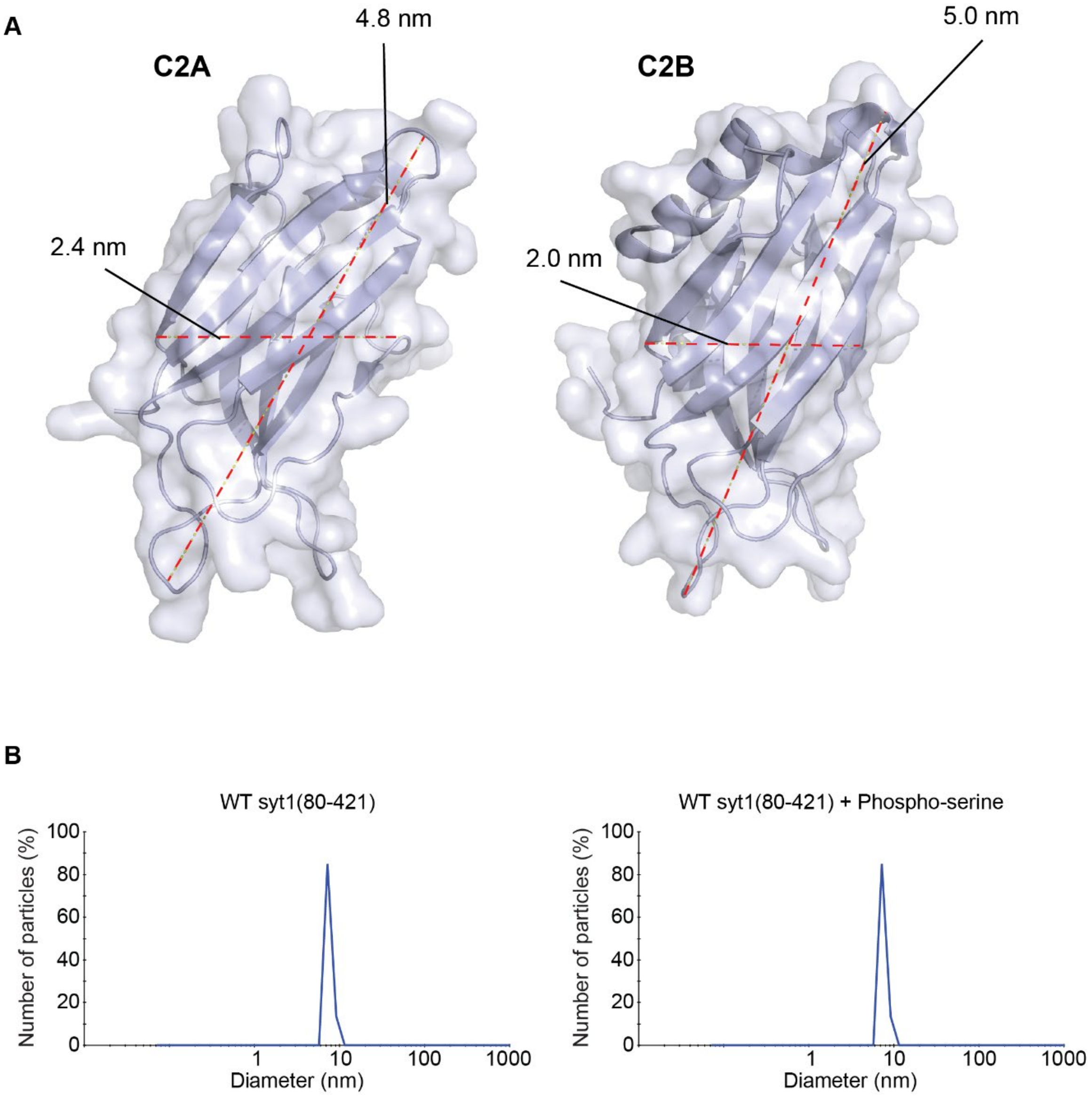

Fig. S1. Dynamic light scattering of the syt1 soluble domain in solution. **A**) Structures of syt1 C2A and C2B domains, with measured dimensions, derived from RCSB PBD files 5t0r and 2yoa, respectively. **B)** Representative dynamic light scatter results of 2 μM WT syt1(80-421) with and without the addition of 100 μM phospho-serine.

A

Recombinant syt1(80-421) juxtamembrane sequences

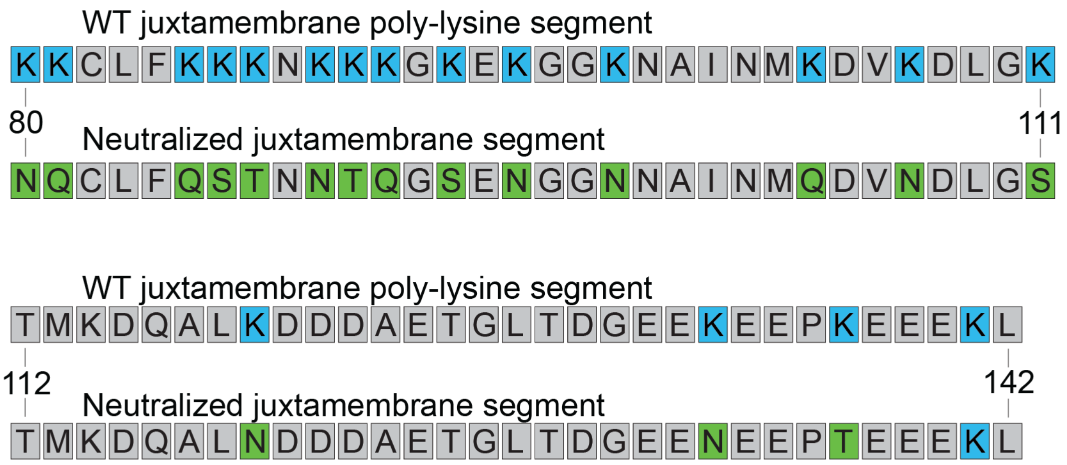

B

Full length syt1 lentiviral expression juxtamembrane sequences

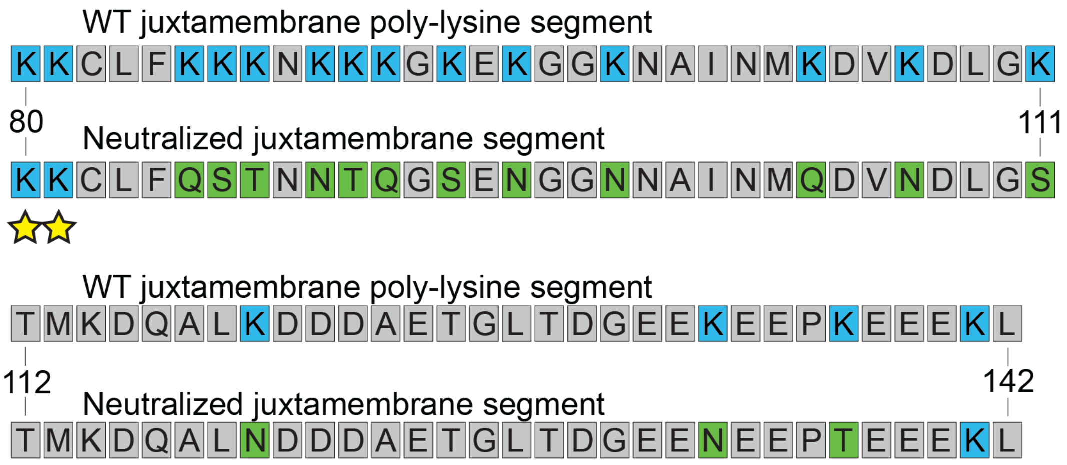

Fig. S2. Complete juxtamembrane amino acid sequences of WT and the Juxta K mutant form of syt1. **A**) For *in vitro* experiments, syt1(80-421) was used. The juxtamembrane sequences of WT and the Juxta K mutant are shown. WT lysine residues are in blue and neutralizing substitutions in the Juxta K mutant are shown in green. **B**) For cell-based experiments of WT and mutant forms of syt1, the full-length protein (residues 1-421) was used. In this case, two lysine resides, 80 and 81, were preserved to maintain proper

topogenesis (1); these are marked with yellow stars. Color-coding is the same as in *panel (A)*.

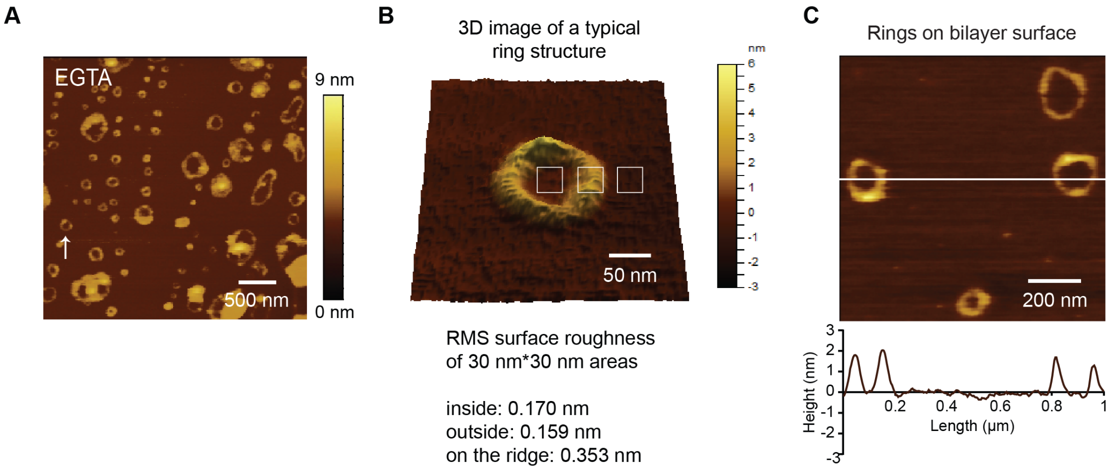

Fig. S3. Syt1(80-421) forms ring-like multimeric structures on the surface of lipid bilayers. **A**) Atomic force microscopy (AFM) topographical images of the entire cytoplasmic domain of syt1 (syt1(80-421)) on a supported lipid bilayer (72% DOPC, 25% DOPS, 3% PIP_2), under aqueous conditions, after a 6-hour incubation. Images were acquired at 9.8 nm/px. A white arrow indicates a representative oligomeric structure with a corresponding zoomed-in three-dimensional representation shown in *panel B*. **B**) Representative 3D image of a ring-like syt1 structure, derived from *panel A*, with surface roughness analysis. Root mean squared (RMS) surface roughness inside (0.170 nm) and outside (0.159 nm) the ring was compared to the roughness of the proteinaceous ring (0.353). **C**) Representative AFM image with topographical line scans across the syt1(80-421) structure that assembled on the surface of the supported lipid bilayer. Line scan shows the height of the internal surface matches the height of the surrounding bilayer (normalized to zero).

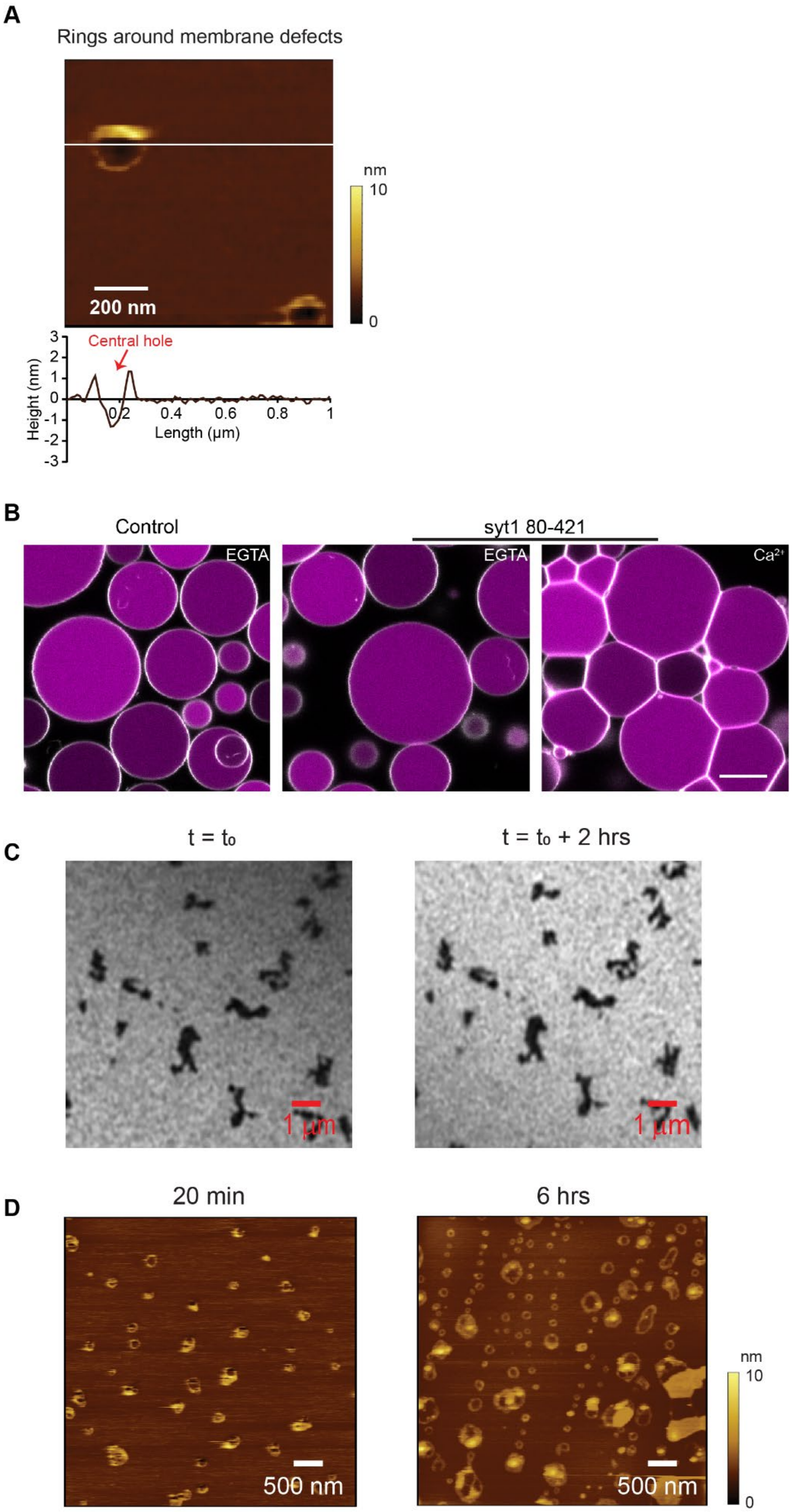

A
Rings around membrane defects
200 nm
nm
10
0
Central hole
Height (nm)
Length (µm)
B
Control
syt1 80-421
EGTA
EGTA
Ca2+
C
t = t0
t = t0 + 2 hrs
1 µm
1 µm
D
20 min
6 hrs
500 nm
500 nm
nm
10
0

Fig. S4. Ring-like syt1 structures around bilayer defects were excluded from analysis. **A**) Representative AFM image with topographical line scans across a syt1(80-421) structure that assembled around a membrane defect. All rings with a central hole were similarly identified by line scanning and were excluded from analysis. **B**) Confocal fluorescence microscopy of giant unilamellar vesicles (labeled with 0.1% Rhodamine-PE, white) composed of DOPC/DOPS (80:20) with encapsulated Alexa-647 dye (magenta). Samples were incubated for 20 minutes with 1 µM syt1(80-421) in 0.5 mM EGTA or 1 mM free Ca^{2+}. Note that after the addition of Ca^{2+}, the vesicles became clustered, due to syt1 bridging between vesicles. Scale bar represents 10 µm. **C**) PC/PS/PIP2 bilayers, containing 0.1% Rhodamine-PE were assembled on freshly cleaved mica discs and imaged by fluorescence microscopy. The stability of defects in the bilayer were assessed by imaging the same region twice, two hours apart. **D**) Representative AFM images of supported lipid bilayers that were incubated with 1 µM syt1(80-421) for twenty minutes or six hours. Note that these images were acquired from independent samples, after syt1(80-421) had been washed away. We found that AFM imaging in the presence of syt1(80-421) greatly affected the AFM tip stability, precluding the ability to visualize the real-time assembly of multimeric structures.

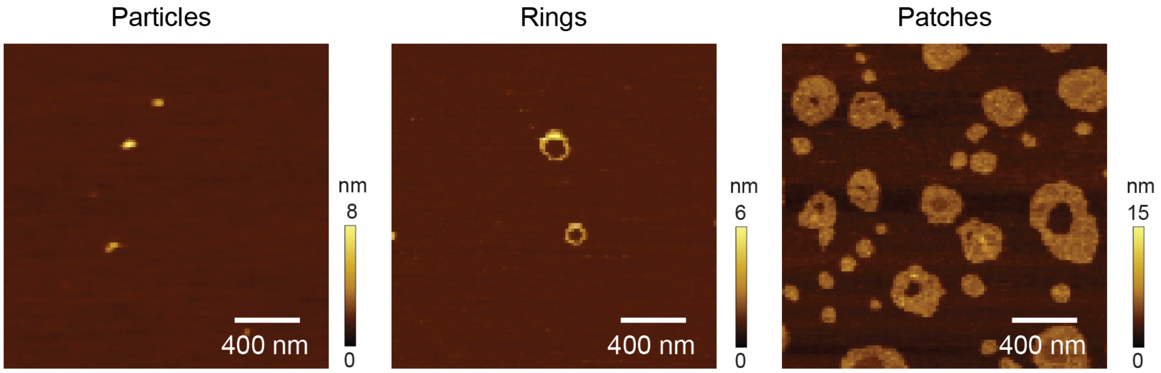

Fig. S5. The morphology of syt1(80-421) structures that assemble on supported lipid bilayers is concentration dependent. **A**) Representative AFM topographical images of syt1(80-421) forming particles (50 nM), rings (1 µM) or patches (3 µM) on supported lipid bilayers.

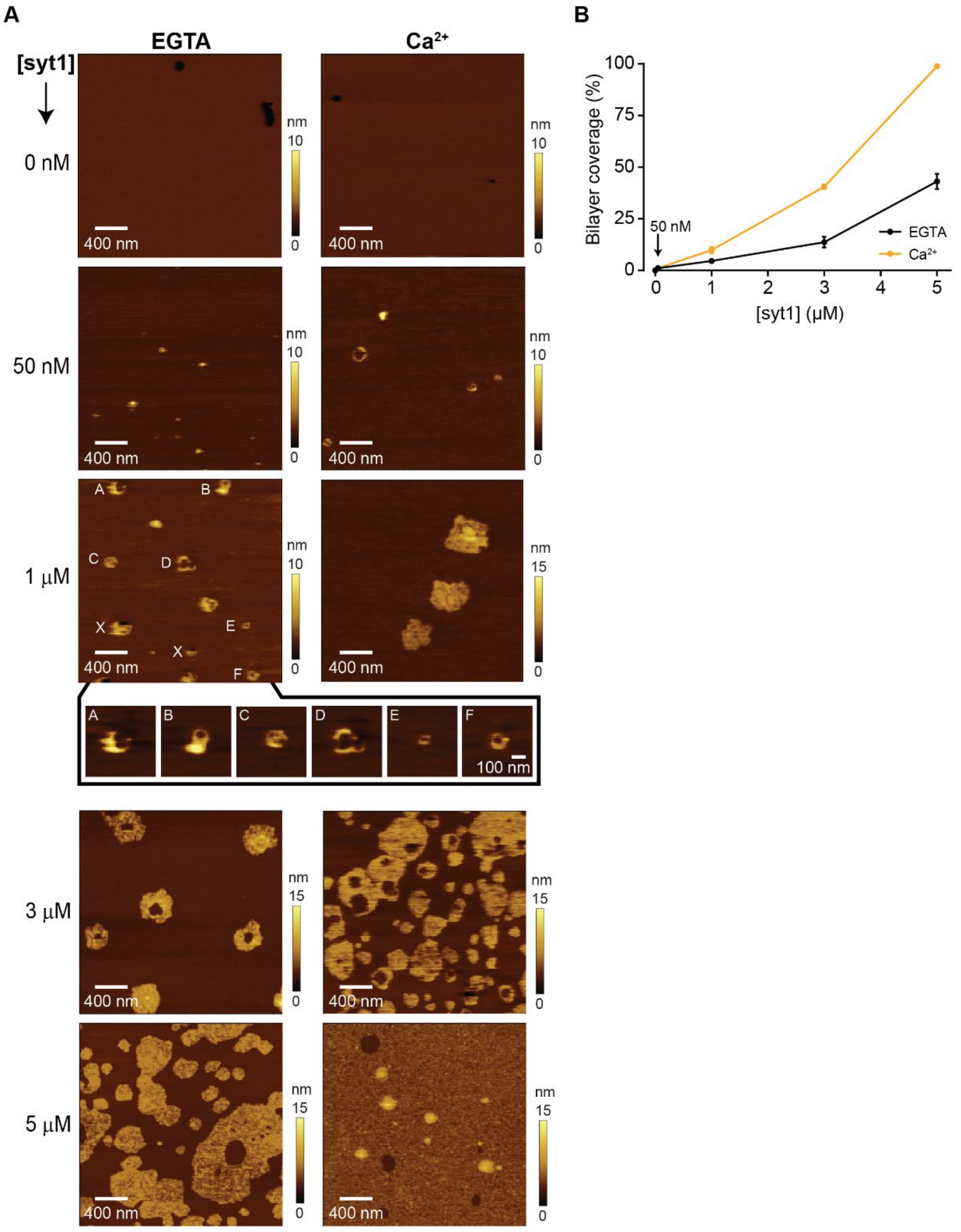

Fig. S6. AFM images of supported lipid bilayers as a function of syt1(80-421) protein concentration. **A**) Representative AFM topographical images of syt1(80-421), at the indicated concentrations, on supported lipid bilayers (72% DOPC, 25% DOPS, 3% PIP_2) in the presence (1 mM) or absence of Ca^{2+} (0.5 mM EGTA). The images were acquired with a resolution of 9.8 nm/px. The inset under the 1□M syt1(80-421) condition in EGTA shows zoomed-in examples, with corresponding annotations, of typical structures. The

white X symbols indicate protein structures surrounding lipid defects, which were excluded from our analysis. **B**) Protein coverage on lipid bilayers, at the indicated [protein], was calculated by setting the height threshold to ≥1 nm above the lipid bilayer surface. The error bars are standard deviation of protein coverage from three different scan areas in the same experiments. **C**) Representative histograms of dynamic light scattering size distributions of syt1(80-421) in solution in the presence or absence (0.5 mM EGTA) of 1 mM free Ca^{2+} and/or 1,2-dihexanoyl-sn-glycero-3-phospho-L-serine (6:0 PS). Note that soluble 6:0 PS alone did not generate a measurable light scatter signal. The experiment was repeated 3 times with consistent results.

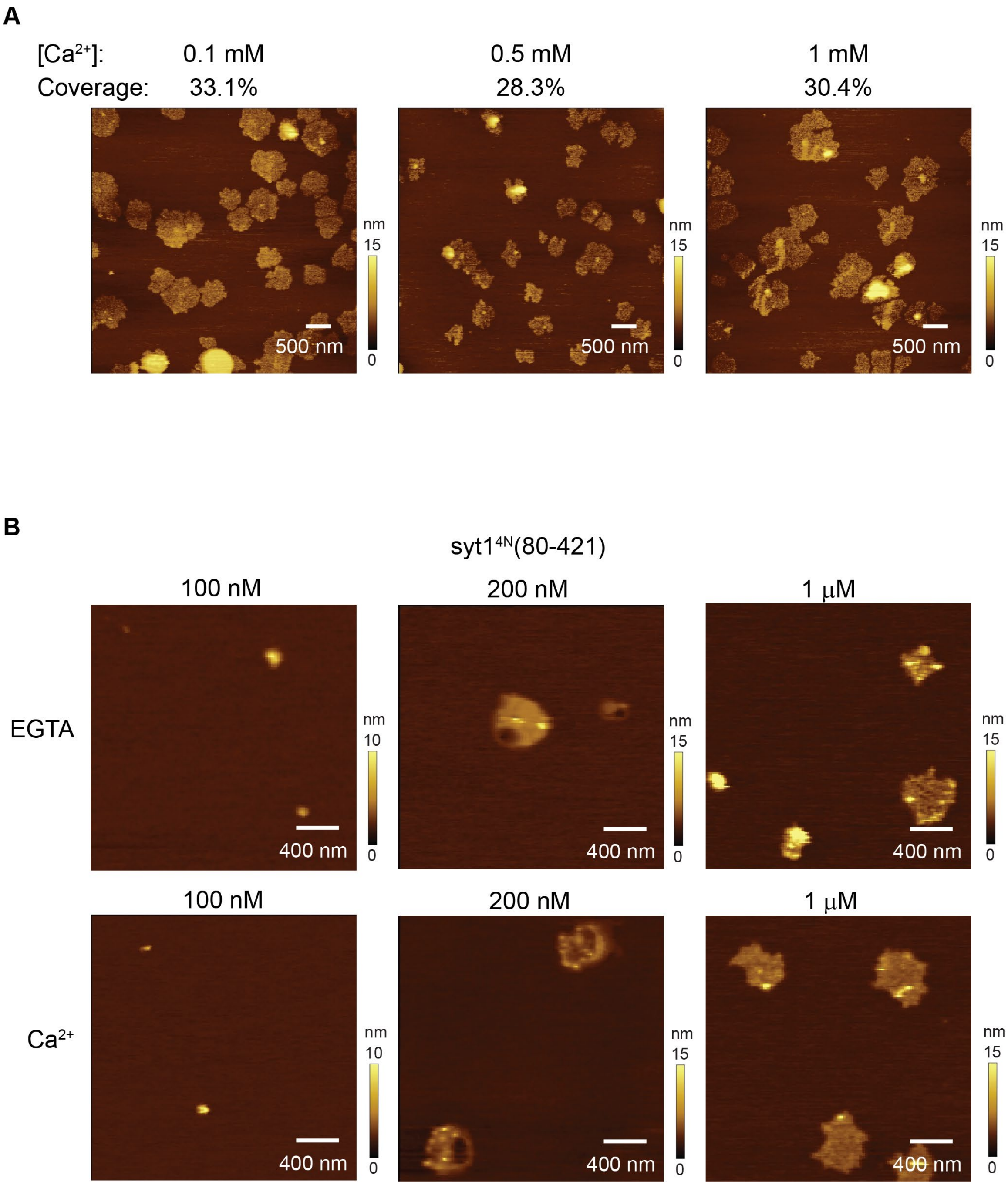

Fig. S7. Ca^{2+} enhances syt1(80-421) self-association. **A**) Syt1(80-421) (1 μM) multimers remained fully formed as patches when the [Ca^{2+}] was reduced from 1 to 0.5 and 0.1 mM. **B**) Substitution of acidic Ca^{2+} ligands in both C2-domains of syt1 prevents Ca^{2+}-induced enhancement of syt1(80-421) oligomerization. Representative AFM imaging of syt1^{4N}(80-421) (D230,232,363,365N) oligomerization on supported lipids bilayers in 0.5 mM EGTA or 1 mM Ca^{2+}.

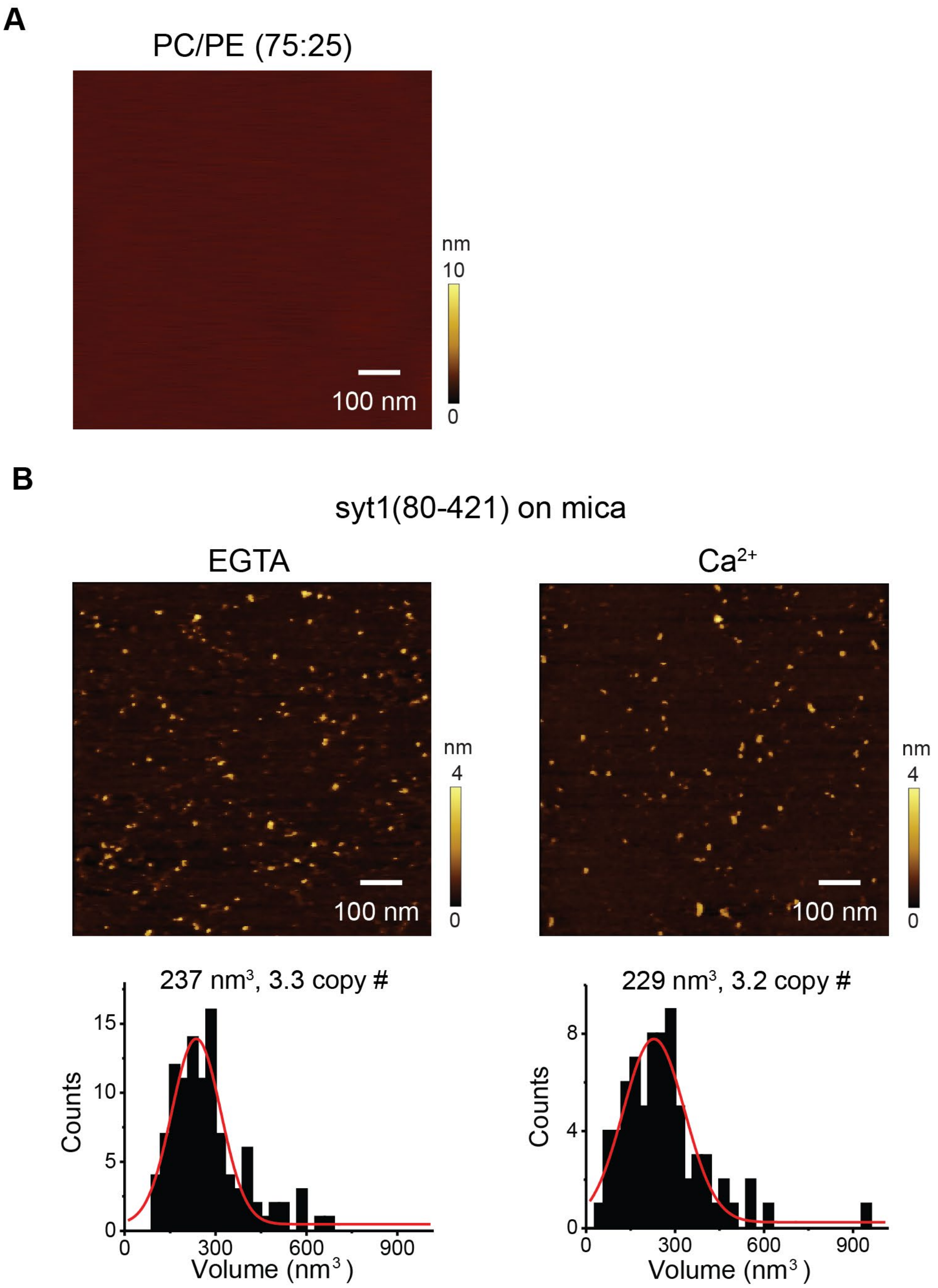

Fig. S8. Syt1 oligomerization on supported lipids bilayers requires anionic phospholipids. **A**) Representative AFM imaging of syt1(80-421) on a supported lipid bilayer composed of PC/PE (75:25). The anionic phospholipids, PS and PIP_2 were substituted with PE to enable efficient rupturing of large unilamellar vesicles on mica to generate the supported lipid bilayers. **B**) AFM imaging of syt1(80-421) in the absence of supported lipid bilayer

(bare mica) in 0.5 mM EGTA or 1 mM Ca^{2+}. Syt1(80-421) size distribution histograms for 0.5 mM and 1 mM Ca^{2+} are shown below.

WT syt1(80-421) oligomeric structures on lipid bilayers

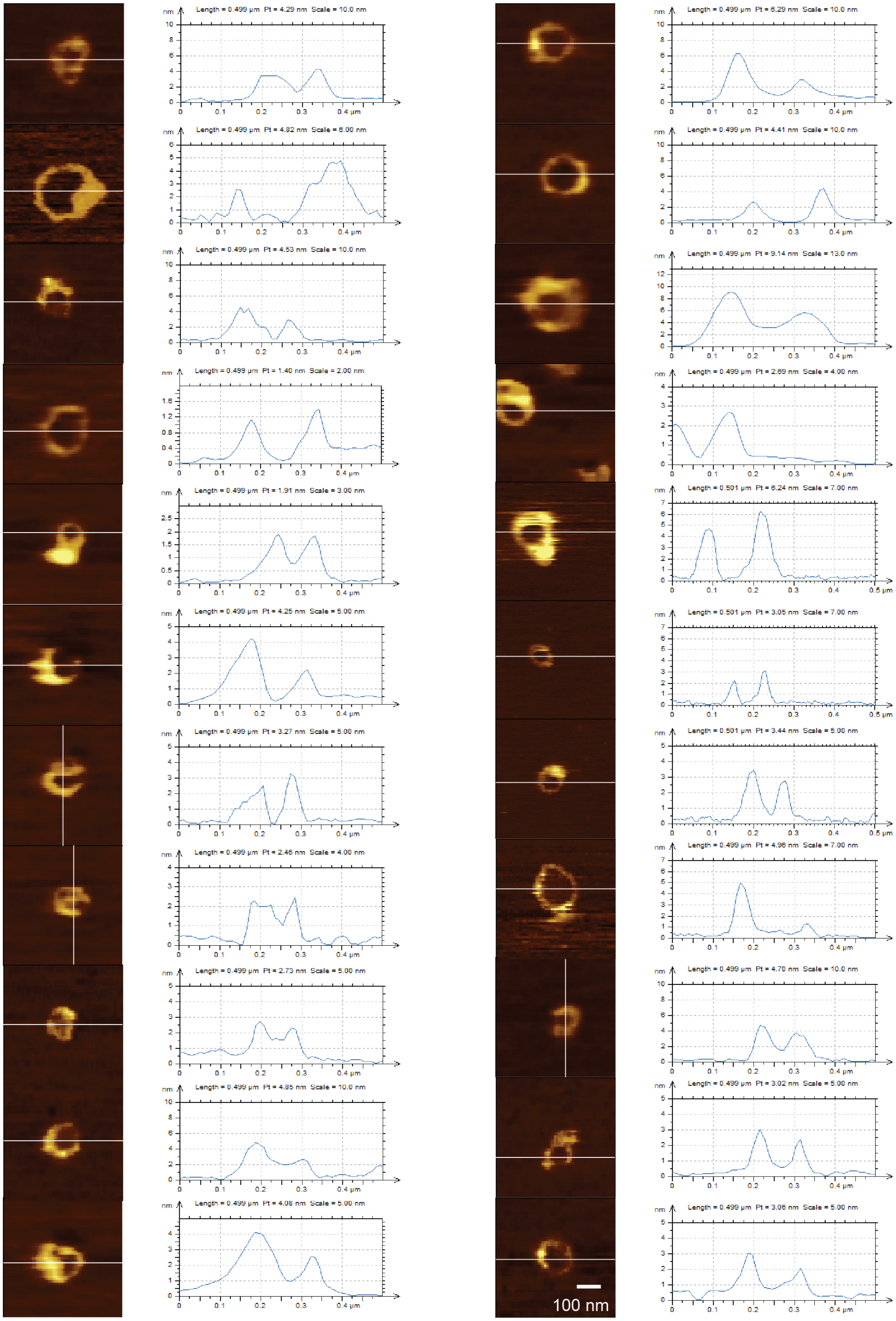

Fig. S9. WT syt1(80-421) forms ring-like structures on supported lipid bilayers in EGTA. Lateral height profiles confirm that only structures lacking a central hole were included in the analysis plotted in Fig. 2D.

K326,327A oligomeric structures on lipid bilayers

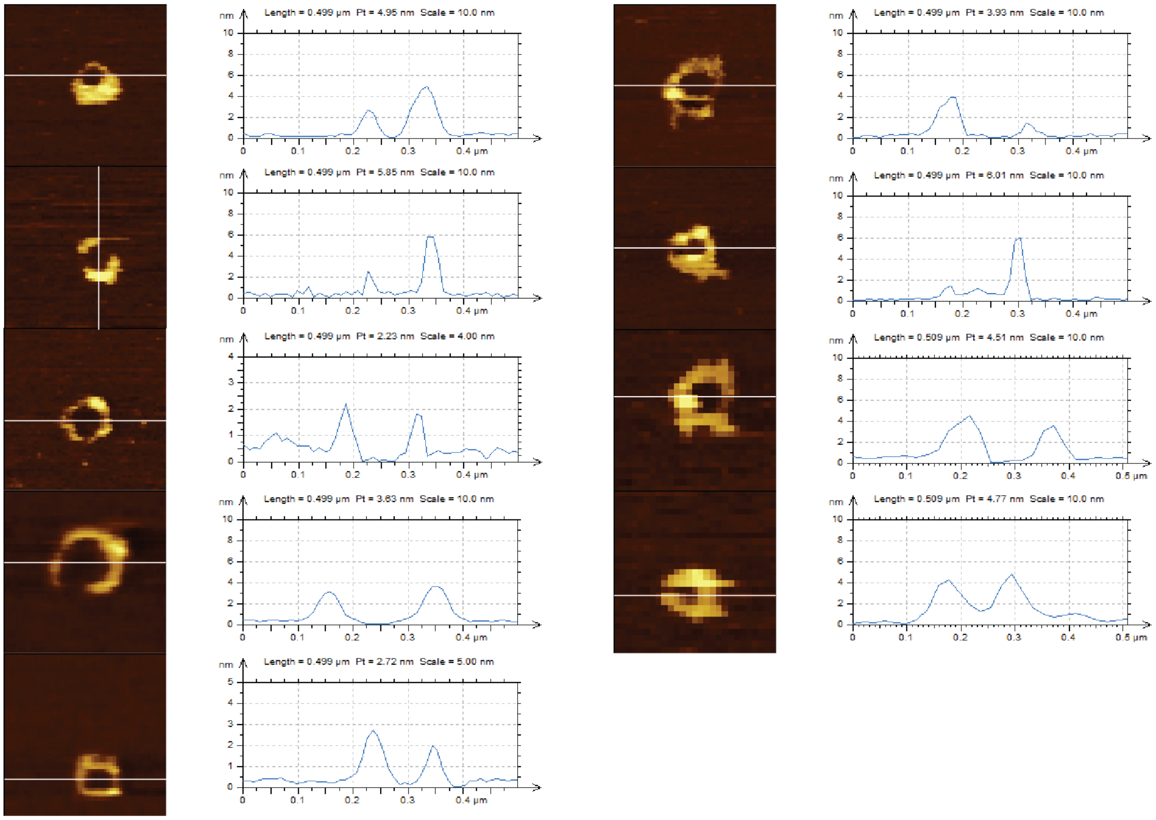

Fig. S10. The K326,327A syt1(80-421) mutant forms ring-like structures on supported lipid bilayers in EGTA. Lateral height profiles confirm that only structures lacking a central hole were included in the analysis plotted in Fig. 2D.

F349A oligomeric structures on lipid bilayers

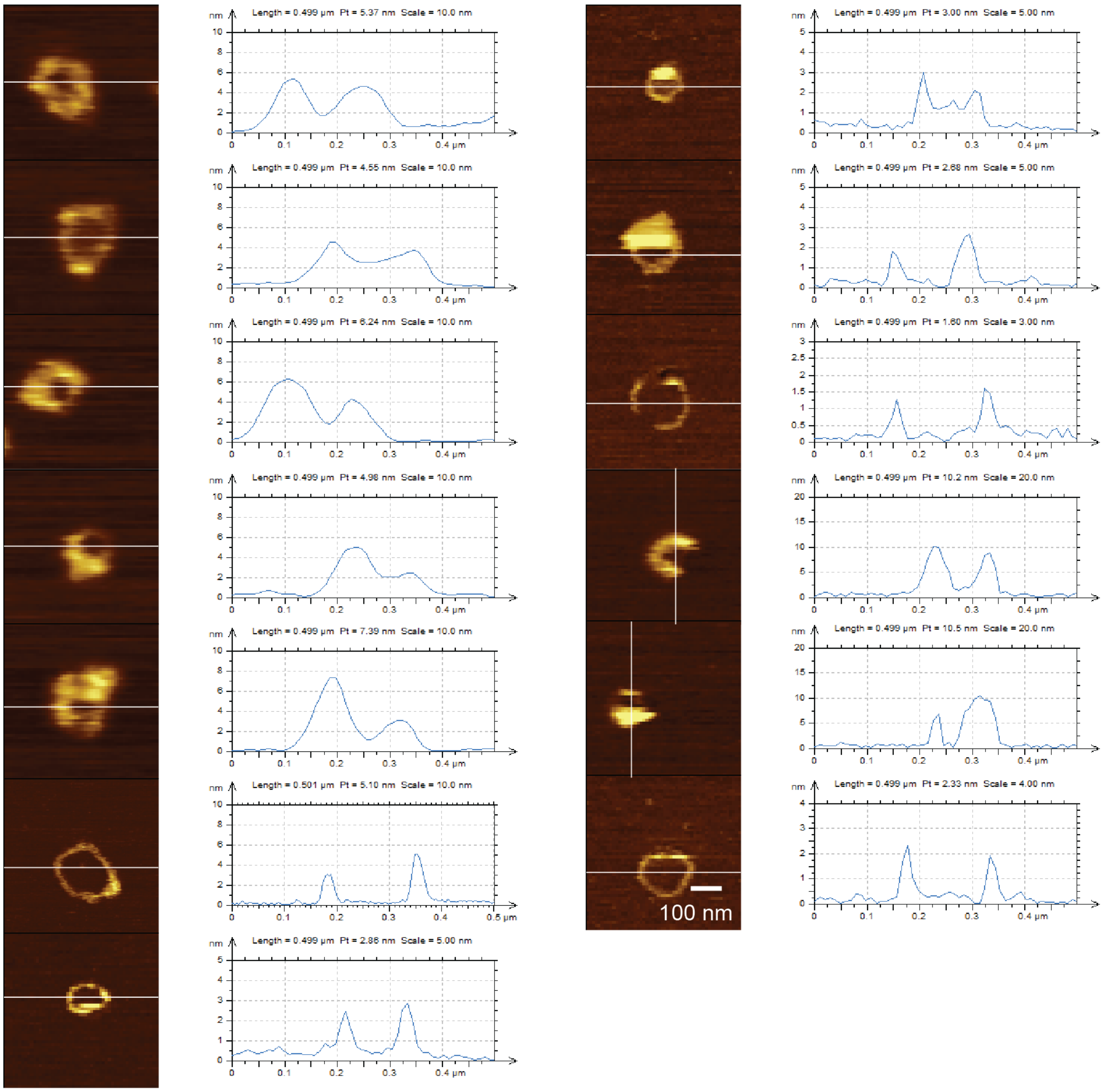

Fig. S11. The F349A syt1(80-421) mutant forms ring-like structures on supported lipid bilayers in EGTA. Lateral height profiles confirm that only structures lacking a central hole were included in the analysis plotted in Fig. 2D.

R398,399Q oligomeric structures on lipid bilayers

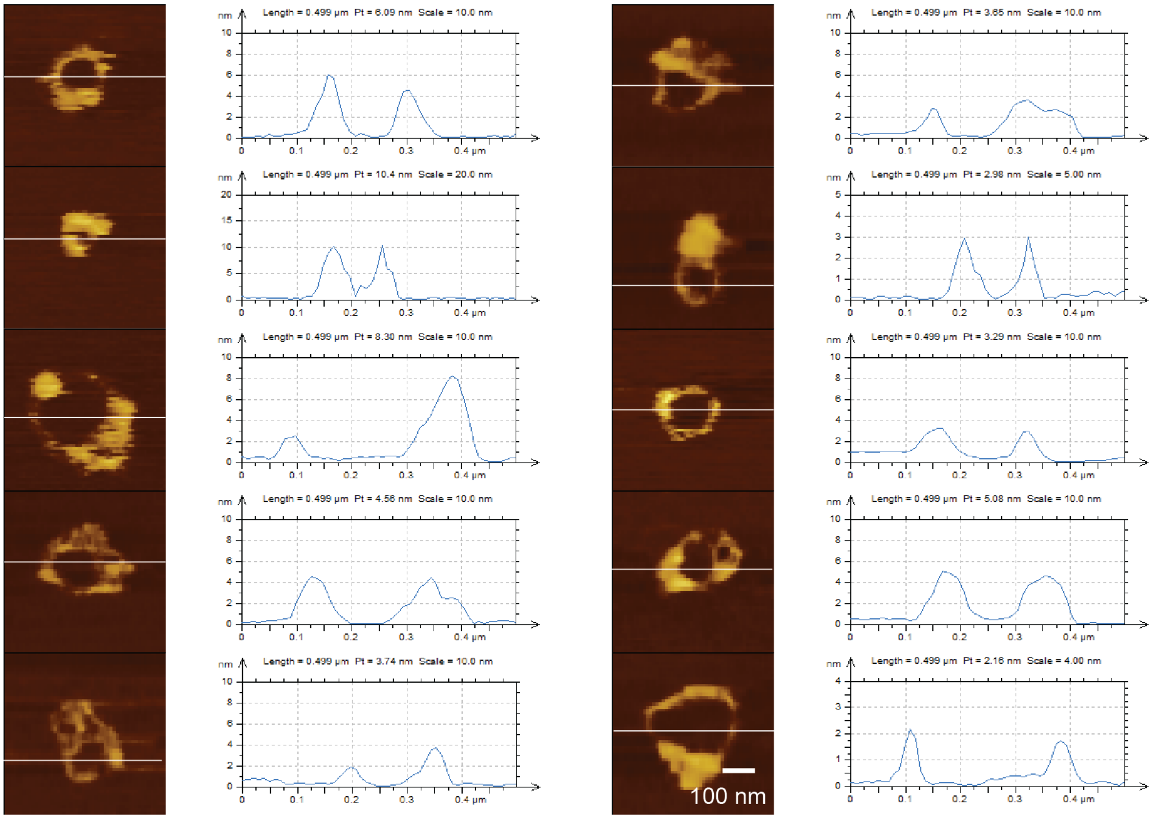

Fig. S12. The R398,399Q syt1(80-421) mutant forms ring-like structures on supported lipid bilayers in EGTA. Lateral height profiles confirm that only structures lacking a central hole were included in the analysis plotted in Fig. 2D.

Juxta K oligomeric structures on lipid bilayers

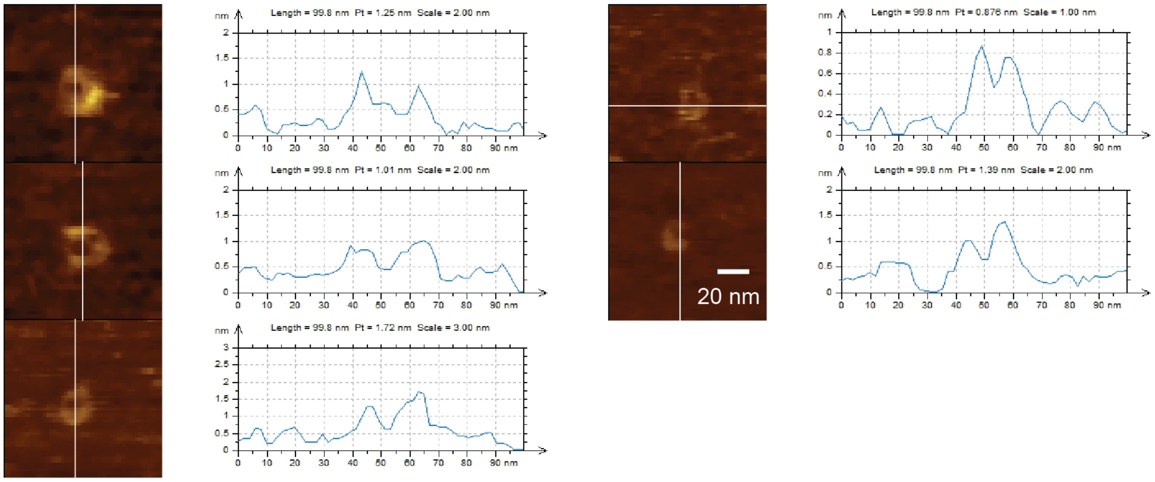

Fig. S13. The Juxta K syt1(80-421) mutant forms ring-like structures on supported lipid bilayers in 1 mM Ca^{2+}. No ring-like structures were observed in EGTA. Lateral height profiles confirm that only structures lacking a central hole were included in the analysis plotted in Fig. 2D.

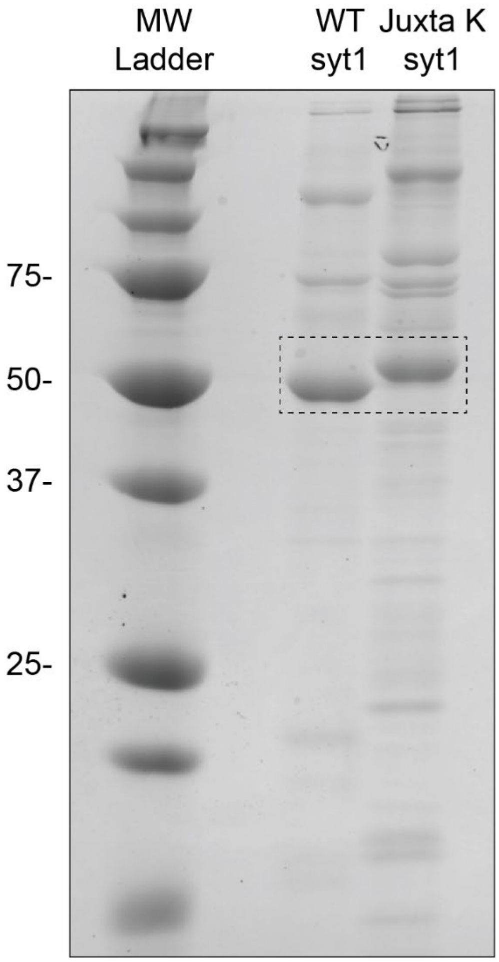

Fig. S14. Recombinant Juxta K mutant syt1 migrates through SDS-PAGE gels slower than WT syt1. Coomassie blue stained SDS-PAGE gel containing a molecular (MW) ladder and recombinant WT or Juxta K mutant syt1 that was purified from *E. coli*. The dotted box indicates the location of the recombinant syt1 protein.

Table S1. Statistics on syt1(80-421) morphology at increasing concentration with and without Ca^{2+}.

Syt1(80-421) concentration (in 0.5 mM EGTA)	30 nM	50 nM	200 nM	1 mM	3 mM
Proportion of particles (%)	100	100	43.3	12.8	0
Proportion of rings (%)	0	0	23.3	78.7	0
Proportion of Patches (%)	0	0	33.3	8.5	100
Ring size (nm)	NA	NA	131±32	175±40	NA

Syt1(80-421) concentration (in 1 mM free Ca^{2+})	30 nM	50 nM	100 nM	1 mM	3 mM
Proportion of particles (%)	40	12.5	22.2	0	0
Proportion of rings (%)	60	62.5	55.6	0	0
Proportion of Patches (%)	0	25	22.2	100	100
Ring size (nm)	147±23	150±31	148±34	NA	NA

Table S2. Numerical values of individual iGluSnFR peaks associated with Fig. 4D.

Condition	Average	SD	N	SEM	Change relative to WT
WT	0.126	0.059	1155	0.001736	0.00 +/- 1.9%
KO	0.011	0.025	212	0.001717	-91.30 +/- 1.4%
KO + WT	0.113	0.053	748	0.001938	-10.30 +/- 2.0%
KO + Juxta K	0.047	0.048	547	0.002052	-62.70 +/- 1.7%
KO + F349A	0.11	0.069	1128	0.002054	-12.70 +/- 2.0%
KO + Juxta K, F349A	0.027	0.038	324	0.002111	-78.60 +/- 1.7%

Supporting Materials and Methods

Reagents.

1,2-dioleoyl-sn-glycero-3-phosphocholine (PC), 1,2-dioleoyl-sn-glycero-3-phospho-l-serine (PS), 1,2-dioleoyl-sn-glycero-3-phosphoethanolamine (PE), 1-palmitoyl-2-oleoyl-sn-glycero-3-phospho-(1'-rac-glycerol) (PG) and 1,2-dioleoyl-sn-glycero-3-phospho-(1′-myo-inositol-4′,5′-bisphosphate) ($PI(4,5)P_2$) were obtained from Avanti Polar Lipids. HEPES was from Fisher Scientific, calcium chloride solution (1.0 M) was from Fluka Analytics, and all other chemicals were from Sigma-Aldrich.

Giant unilamellar vesicle and supported lipid bilayer microscopy

Giant unilamellar vesicles (GUVs) and rhodamine-PE labeled supported lipid bilayers (SLB) were imaged using a Zeiss 880 Airyscan microscope. GUVs were prepared by drying 15 μl of 1 mM DOPC/DOPS lipids, plus 0.1% rhodamine-PE in three locations onto indium tin oxide coated glass slides and then performing electroformation with 10 Hz, 4 V alternating current for two hours in 1 mM HEPES, 200 mM Sucrose solution. The buffer also contained 10 μM Alexa-647 dye. The excess dye was removed by filtration. The SLBs were prepared on mica discs as described above, except with the inclusion of 0.1% rhodamine-PE within the lipid mixture.

Cell Culture

Syt1 floxed neurons were prepared as previously described (2). Briefly, hippocampal neurons were dissected at P0 from syt1 floxed mouse strain (Quadros et al., 2017), trypsinized (Corning; 25-053-CI), triturated, and plated on glass coverslips (Warner instruments; 64-0734 (CS-18R17)) coated with poly-D-lysine (Thermofisher; ICN10269491) and EHS laminin (Thermofisher; 23017015). Neurons were grown for at least 14 days in Neurobasal-A (Thermofisher; 10888-022) medium supplemented with B-27 (2% Thermofisher; 17504001), Glutamax (2 mM Gibco; 35050061), and pen/strep before experiments. For virus preparation, HEK293T cells (ATCC) were cultured following ATCC guidelines and were tested for mycoplasma contamination using the Universal Mycoplasma Detection Kit (ATCC; 30-1012K), and validated as HEK293T cells using Short Tandem Repeat profiling by ATCC (ATCC; 135-XV) within the last year.

Lentivirus production and use

Lentivirus production was performed as described previously (2). Syt1 expressing lentiviral constructs were subcloned into the FUGW transfer plasmid (FUGW was a gift from David Baltimore (Addgene plasmid # 14883) (3). Our lab has previously modified this construct, replacing the ubiquitin promoter with the human synapsin I promoter (4). Lentivirus expressing Cre was added to neuronal cultures at day one in-vitro (DIV), iGluSnFR and pHluorin constructs were also added at 1 DIV. Syt1 constructs were added at 5 DIV.

Plasmid construction for lentiviral expression

All plasmids, unless otherwise noted, were constructed using our lab's modified lentivirus backbone of choice derived from FUGW. The glutamate sensor is the same as used previously (2). The pHluorin construct used here was subcloned into our modified FUGW transfer vector from the original vGlut1-pHluorin construct (5). All synaptotagmin 1 constructs were subcloned into our modified FUGW transfer plasmid from their original bacterial expression plasmids. For Cre expression, we used the transfer plasmid pLenti-hSynapsin-CRE-WPRE (pLenti-hSynapsin-CRE-WPRE was a gift from Fan Wang (Addgene plasmid # 86641) (6).

Immunoblot analysis

Immunoblots were performed as described previously (2). Primary antibodies were: anti-syt1 (1:1000, 48) (lab stock; mAB 48; RRID:AB_2199314). Secondary antibodies were: goat anti-mouse IgG2b-HRP (Biorad, M32407; RRID:AB_2536647).

Immunocytochemistry (ICC)

Immunocytochemistry was performed as previously described (2). Primary antibodies were: anti-syt1 (1:100, 48) (lab stock; mAB 48; RRID:AB_2199314) and anti-synaptophysin (SYP) (1:500) (SySy; 101 004; RRID:AB_1210382). Secondary antibodies used were: goat anti-guinea pig IgG-Alexa Fluor 546 (1:500) (Thermofisher; A-11074, RRID:AB_2534118) and goat anti-mouse IgG2b-Alexa Fluor 647 (1:500) (Thermofisher; A-21242; RRID:AB_2535811). Images in Fig. 3 were acquired on a Zeiss LSM 880 with a 63x 1.4 NA oil immersion objective using the Airyscan super-resolution detector. The same laser and gain settings were used for each condition. Images were deconvolved using automatic Airyscan settings and the same linear brightness and contrast adjustments were applied to all images for figure presentation.

Statistics

Values from analysis and number of trials (n) for each experiment are listed in the figure legends. All analysis was done using GraphPad Prism 7.04 (GraphPad Software Inc).

1. Y. Kida, M. Sakaguchi, M. Fukuda, K. Mikoshiba, K. Mihara, Membrane topogenesis of a type I signal-anchor protein, mouse synaptotagmin II, on the endoplasmic reticulum. *Journal of Cell Biology* **150**, 719-729 (2000).
2. J. D. Vevea, E. R. Chapman, Acute disruption of the synaptic vesicle membrane protein synaptotagmin 1 using knockoff in mouse hippocampal neurons. *Elife* **9**, 24 (2020).
3. C. Lois, E. J. Hong, S. Pease, E. J. Brown, D. Baltimore, Germline transmission and tissue-specific expression of transgenes delivered by lentiviral vectors. *Science* **295**, 868-872 (2002).
4. S. Kugler, E. Kilic, M. Bahr, Human synapsin 1 gene promoter confers highly neuron-specific long-term transgene expression from an adenoviral vector in the adult rat brain depending on the transduced area. *Gene Therapy* **10**, 337-347 (2003).
5. S. M. Voglmaier *et al.*, Distinct endocytic pathways control the rate and extent of synaptic vesicle protein recycling. *Neuron* **51**, 71-84 (2006).
6. K. Sakurai *et al.*, Capturing and manipulating activated neuronal ensembles with CANE delineates a hypothalamic social-fear circuit. *Neuron* **92**, 739-753 (2016).
